# The Influence of College Students’ Innovation and Entrepreneurship Intention in the Art Field of Art Film and Television Appreciation by Deep Learning Under Entrepreneurial Psychology

**DOI:** 10.3389/fpsyg.2022.900176

**Published:** 2022-06-09

**Authors:** Minxin Wang, Yefan Shao, Shiman Fu, Lele Ye, Hongming Li, Guodong Yang

**Affiliations:** ^1^Development and Planning Division, Wuhan Business University, Wuhan, China; ^2^Advertising Institute, Communication University of China, Beijing, China; ^3^Southampton Education School, University of Southampton, Southampton, United Kingdom; ^4^Zhijiang College of Zhejiang University of Technology, Shaoxing, China; ^5^College of Public Management and Liberal Arts, Dalian Maritime University, Dalian, China

**Keywords:** deep learning, film and television education, innovation and entrepreneurship, values cultivation, entrepreneurial psychology

## Abstract

There are many films and televisions (FATs) on the Internet, but the quality is uneven. This study explores the ability of college students to screen good films and resist bad films in television works in such a large environment. In the deep learning model of FAT, the ability of college students to think about the ideas expressed and the degree of influence on college students’ values are analyzed. Based on this conceptual basis, a questionnaire is designed for the intention and influencing factors of college students’ FAT innovation and entrepreneurship. It reflects the influence of concentration on FAT learning, the cognitive level of deep learning, the ability to process deep learning ideas, the feeling of the teaching process, and the process of self-learning, which all positively impact college students’ FAT entrepreneurial intentions. The importance of innovative deep learning is highlighted, which proves that a good deep learning course guidance method can improve students’ interest and ability and provide a reference for relevant colleges and universities to cultivate pertinent talents of the field of FAT.

## Introduction

Film and television (FAT) and animation are some of the newest industries in the 21st century. Most college students of this generation grew up under the influence of this FAT culture. With the development of media technology, FAT has become one of the necessities of life for college students (Vaterlaus et al., 2019), which has had a transformative impact on college students’ values, aesthetic ability, and various behaviors. But FAT culture is also a double-edged sword. With the current popularity of short videos, some wrong values can also hurt college students (Dalpiaz and Cavotta, 2019). Entrepreneurship in FAT is also a preferred project for graduate entrepreneurship for many college students. Therefore, how to extract the essence and discard the dross from many videos, help college students to form correct FAT entrepreneurial values, and form a positive FAT entrepreneurial intention is an indispensable topic of discussion (Shankar, 2019).

As one of the most common information dissemination media in people’s life, FAT culture will have a very significant impact on people’s inner values. Research has found that because children or young people have very sensitive minds, videos and animations of all kinds can affect their behavior and consciousness. Therefore, FAT works with positive values that need to be screened (Sopekan et al., 2020). Whether it is to cultivate the correct direction of contemporary college students’ thinking or to establish the image of the country’s FAT culture, it is necessary to use the information dissemination ability of FAT culture correctly and reasonably. It is subsequently pointed out that popular culture as a distinct cultural form has its own historical, artistic, and textual categories, but which may have evolved with the rise of various cultures (Danesi, 2019). Therefore, it is very important to cultivate the correctness of FAT works. In the group of college students who intend to use the FAT field as entrepreneurial students, some scholars believe in understanding the current situation of learners’ use of social media in entrepreneurship courses. They should explore the use and satisfaction of social media in entrepreneurship courses from learners’ perspectives. The results reveal that trust, profit, learning, and socialization are the three elements that satisfy the learning psychology, especially the trust element (Wu and Song, 2019). Innovative education must improve the psychological quality of students. In addition, research suggests that narcissism and competition predict weak self-esteem, while admiration predicts optimal self-esteem. The competition is between narcissism and admiration. This supports its positioning in the self-importance dimension of the narcissistic spectrum model (Radoslaw et al., 2018). In the study of psychological state, it is believed that narcissism or spiritual quality has a certain influence on educational entrepreneurship and practice (Wu et al., 2019). In the educational exploration of entrepreneurship, it is believed that a scientific and reasonable entrepreneurship curriculum system is essential for effective entrepreneurship education. Encouraging students to engage in an ongoing entrepreneurial process through entrepreneurship courses is beneficial to the overall development of the economy and society (Su et al., 2021). Therefore, it is an important task to investigate college students’ intention to start a business in the FAT field in a deep learning environment.

This study studies the impact of college students in the FAT field and their innovation and entrepreneurial intentions. Firstly, current undergraduate deep learning models are introduced. Secondly, the influence of animation on college students’ values is outlined and discussed. On this basis, the questionnaire survey method is adopted to investigate the innovation and entrepreneurship intentions of college students. A total of 100 questionnaires are distributed to universities in a certain place. After the survey, a total of 92 questionnaires are returned. Among them, the number of valid questionnaires was 79. The results of the questionnaires are sorted and analyzed, and the results of the questionnaire validity test were about to 86%, indicating good validity. In addition, college students’ FAT entrepreneurial intentions are explored, and some conclusions are drawn. These data provide a certain direction for college students’ entrepreneurial guidance. The novelty lies in using deep learning as a learning model to explore the factors that stimulate college students’ FAT entrepreneurial psychology.

## Materials and Methods

### Deep Learning Methods for Film and Television Appreciation Education

**FIGURE 1:**
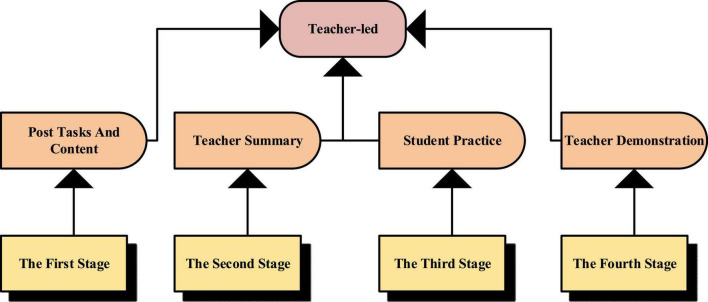
The traditional shallow learning process.

In the early days, deep learning was a learning model for artificial intelligence (AI) deployed in the field of AI. It extracts various neural network information that can be expanded from various learning features to form a multi-dimensional learning relationship by evolving the powerful capabilities of computers and algorithms to achieve deep conceptual machine cognition (Gibbons et al., 2019). Compared with traditional shallow learning, the deep learning method understands the concept of learning more deeply to explore the subsequent expansion problems. With the continuous improvement and development of deep learning, it has also been used to represent deep learning which is different from shallow learning (Tian et al., 2019). Currently, the widely used traditional shallow learning process is shown in Figure 1.

**FIGURE 2:**
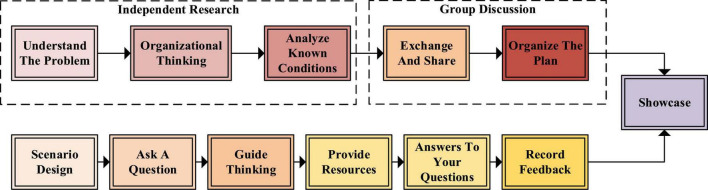
The flow of new deep learning.

In Figure 1, in this learning mode, teaching is a process in which teachers demonstrate and students imitate. A process is repeated by correcting mistakes. After a long time, students will naturally have boring psychology. Although this approach is more rigorous and unified, it ignores the individual differences and personality development of students. Lack of interaction between teachers and students can lead to low learning interest (Wei et al., 2019). Therefore, the novel deep learning process is shown in Figure 2.

In Figure 2, this deep learning model mainly takes students as the main body of the classroom, rather than the content of the classroom dominated by the teacher alone. Compared with the traditional situation where one teacher faces dozens of students simultaneously, deep learning pays more attention to dividing students into groups for study and discussion. Therefore, the differences between the two learning modes are shown in Table 1.

**TABLE 1:** Differences between deep learning and shallow learning.

Method	Deep teaching method	Shallow teaching method
Form	Group learning	Class
Object	Student-centered	Teacher-centered
Measure	The focus is on developing skills	The focus is on transferring knowledge
Interactive	Student interaction	Lack of interaction with students
Object	Students and teachers work together	Teacher’s one-person show
Error correction	Summarize from mistakes	Lack of self-reflection
Task	Teachers are only responsible for posting tasks	Teachers take full responsibility
Focus	Long-term memory	Weak memory of knowledge
Expand	Students can make use of the knowledge and resources they have acquired	Learning tasks are the only resource

**FIGURE 3:**
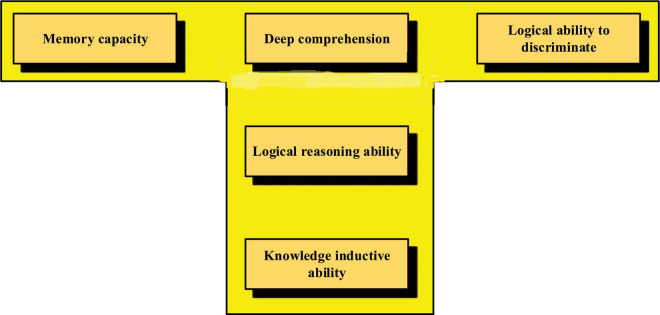
Demonstration of deep learning capabilities.

In this deep learning process, students can improve their self-evaluation level through metacognitive ability and expand the information they have learned. In addition, the ability to communicate and cooperate actively is reflected in communicating actively and cooperating with others in classroom autonomous exploration activities, after-class practical activities, online media, and other activity venues (Zhou et al., 2021). Therefore, the integrated learning ability of deep learning is reflected in the five aspects as shown in Figure 3, which is the T-shaped thinking commonly used in deep learning.

In Figure 3, creative practice ability is reflected in innovative ideas and specific operation ability. The deep integration of innovative thinking and practical operational ability achieves the best realization of thinking and practice. The level of learning empathy experience is reflected in the ability to integrate learning content and learning emotion. In the process of receptive learning and autonomous learning, students fully understand and think about the learning content, discover their ambition and interest in self-learning, and realize the self-fulfilling learning state (Hassan, 2020).

### The Influence of Film and Television Animation on the Entrepreneurial Values of College Students

Film and television culture emerged with the development of science and technology in the 20th century. At present, the definition of FAT culture is divided into three levels: material culture, institutional and institutional culture, and art as a product of the human spirit (Solanas and Getino, 2021). From the perspective of cultural form, FAT culture is a comprehensive culture involving various social sciences and humanities such as sociology, psychology, aesthetics, art and art, and communication. From the perspective of cultural value orientation, FAT culture embodies the synthesis of high art and popular art (Atabek and Nurnazar, 2021). The characteristics of FAT animation are shown in Table 2.

**TABLE 2:** Characteristics of FAT animation.

FAT features	Feature performance
Immediacy	FAT animation generally transmits information directly through images and sounds, connecting human eyes and ears with the boss, reflecting objective real-time. The person captures the key points of the information and makes it clear immediately.
Universality	Universality means that the threshold for dissemination of FAT animation has become lower. If the illiterate cannot understand textual information, then FAT animation can allow people with low education levels to obtain the same amount of information as ordinary people. Therefore, the film is a great example of people of different cultures and ages being able to get the same message from a movie.
Entertainment	People love movies, animations, and short videos because they all share a common feature, that is, entertainment. Entertainment is common sense in all TV programs. All of them are designed to provide entertainment for the audience.
Introductory	Since FAT are artificially short, the subjective intention of the photographer can be used to convey the information they want to express to the audience. Just like various patriotic movies, they can convey the emotions of patriotism and self-improvement to the audience. Therefore, whether it is TV movies or variety shows is a kind of orientation for people.

**FIGURE 4:**
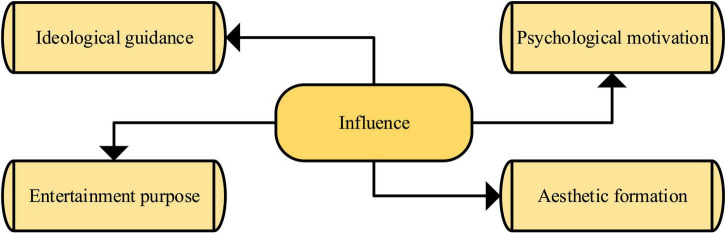
The impact of FAT on college students.

Film and television will have different effects on college students. Outlook on the world, life, and values are the most fundamental contents of Ideological and Political Education (IPE). Values education is an important part of IPE. College students are in an important period of value formation and stabilization and are easily affected by external things (Astuti et al., 2019). FAT culture has penetrated the life of college students, which has a subtle, profound, and lasting impact on their values of college students. Four significant effects of FAT on college students are shown in Figure 4.

In Figure 4, contemporary college students can access and watch a huge number and variety of FAT works. However, the production level of these FAT works is uneven, and a large amount of bad information is wrapped in them, bringing new problems and challenges to the value of education of college students. The best way to deal with this challenge is to use FAT culture as the carrier and to use the characteristics of FAT culture to educate college students on values that are rich in content, flexible in form, and attractive.

**FIGURE 5:**
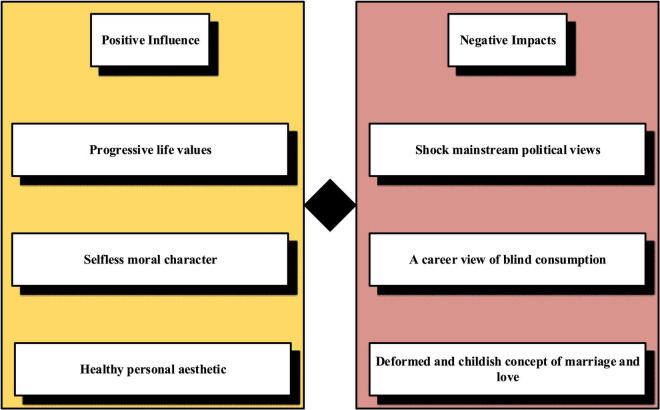
Positive and negative impacts.

Film and television culture exerts a double influence on the values of college students. It enables the confused college students to establish lofty ideals and find the right direction in life. It can also dispel the correct values transmitted by school and family education and make college students go astray. From the aspects of life, moral, aesthetic, political, marriage and love, consumption values, etc., the positive and negative effects of FAT culture on college students’ values are specifically analyzed, and the reasons for the negative effects are discussed (Rustamova, 2019). The division of positive and negative effects of FAT culture on college students’ values is not absolute. For example, FAT culture also positively impacts college students’ values of marriage and love, but it can better reflect the real problems of today’s society. Therefore, the influence of FAT culture on college students’ psychological mobilization and values generally has positive and negative effects. Among them, positive and negative effects are generally divided into the following categories, as shown in Figure 5.

In Figure 5, at present, many FAT works are shoddy and vulgar in quality and content under the persuasion of capital and economic interests. In this situation, relying solely on the self-discipline of FAT culture creators cannot be perfected. The government must strengthen the supervision and management of the production and dissemination of FAT culture, use a good system to encourage the producers of excellent works to give full play, and make the creators and distributors of bad FAT unprofitable (Newsinger and Eikhof, 2020).

The value of education of colleges and universities plays a decisive role. Successful value education in colleges and universities can enable college students to form immunity to negative information and minimize the negative impact through rational value judgment and selection ability when facing the negative information of FAT culture (Yüzgeç and Sütçü, 2020). Compared with the strong influence of today’s FAT culture, the value of education in colleges and universities is increasingly showing the weakness of insufficient effectiveness.

The reasons for the students themselves are also very important. The lack of FAT appreciation ability highlights the lack of value selection and judgment ability of college students. College students neglect some works with high ideological and artistic content due to a lack of attention and correct guidance. Some bad ideological tendencies in the works cannot be identified and effectively criticized by college students (Wu, 2021). There is often a misalignment between FAT dramas with intrinsic value and the actual acceptance ability of college students.

### Questionnaire Design on the Mobilization of College Students’ Entrepreneurial Psychological Willingness by Film and Television

As a measure that can intuitively reflect various data indicators of experimental subjects, questionnaires are used in many social or school phenomena. Therefore, this study adopts the questionnaire method, combines the concepts of in-depth learning of FAT education and the psychological impact of FAT on students’ innovative and entrepreneurial intentions, and designs a questionnaire on the influencing factors of FAT on the entrepreneurial intention for college students. The questionnaire adopts a five-point system. The design content is shown in Table 3.

**TABLE 3:** Questionnaire on the psychological entrepreneurial intention of FAT.

Basic information			
Your major: A. Science engineering B. Liberal arts Your professional name:	
Your gender: A. Male B. Female			
Your grade: A. Freshman B. Sophomore C. Junior D. Senior			
Number Contents		Options and levels A. Strongly agrees B. Comparative identification C. Neutral view D. Do not agree E. Strongly opposed
**The impact of FAT learning focus**			
1	Before the class, I will pre-watch the FAT I have learned.	A. B. C. D. E.
2	I often ask teachers about the information expressed in the FAT	A. B. C. D. E.
3	I often watch clips from movies repeatedly	A. B. C. D. E.
4	I will watch related works like the FAT.	A. B. C. D. E.
**Deep learning cognitive level**			
5	I will use different viewing standards for different FAT works	A. B. C. D. E.
6	When I feel confused about the information in the film, I think for myself first	A. B. C. D. E.
7	I will appreciate a piece of FAT works in the way I plan	A. B. C. D. E.
8	I will regularly think about the ideas expressed in FAT works	A. B. C. D. E.
**Deep learning thought processing ability**			
9	I can always understand the main idea expressed in FAT works	A. B. C. D. E.
10	I can use my way of thinking or map to describe the language of FAT	A. B. C. D. E.
11	When I watch new films and television works, I can generate new ways of thinking	A. B. C. D. E.
12	I can always keep a clear memory of the main idea of the FAT	A. B. C. D. E.
Experience of teaching			
13	Teachers always use unusual ways to get me interested in learning	A. B. C. D. E.
14	Compared with memory, teachers’ pay more attention to making students understand the main idea	A. B. C. D. E.
15	Teachers will accept students’ suggestions and improve teaching methods	A. B. C. D. E.
16	Teachers often encourage students to participate in activities that create videos	A. B. C. D. E.
**Self-learning process**			
17	I always feel a deep sense of satisfaction	A. B. C. D. E.
18	After-school work on FAT makes me anxious	A. B. C. D. E.
19	All classes are fun for me	A. B. C. D. E.
20	I think watching movies and TV works after class can improve one’s mind	A. B. C. D. E.
**Others**			

After the questionnaire is designed, it needs to be tested for reliability. In the reliability analysis of the questionnaire, the higher the reliability of the questionnaire, the more accurate the data can represent the reality the higher the reliability of the questionnaire. In the analysis method of reliability, Cronbach’s α, which is the most versatile, is used to calculate its reliability (Shrestha, 2021), as shown in Eq. 1:


(1) $$A\,l\,p\,h\,a=\left(\frac{k}{k-1}\right)\,\left(1-\frac{\sum_{i=1}^{k}S_{i}^{2}}{S_{x}^{2}}\right)$$

*k* represents the total number of items on the scale. $S_{i}^{2}$ represents the score variance of the *i*-th item. $S_{x}^{2}$ represents the variance of the total number of questions. Cronbach’s α can be used to measure unity in attitude and opinion tables.

Structural equation modeling is an algorithm that has a verification function. It is an extension of the general linear model, which can make up for the shortcomings of traditional statistics and can analyze multiple variables and results. The structural equation model generally requires that the sample size is greater than 100. The verification effect is best if the sample size exceeds 200 to satisfy *N*/*P* > 10 and *N*/*t* > 5. *N* is the total sample size. *t* is the number of free parameters, and *P* is the total observed variable. The structural model is shown in Eq. 2:


(2) η=β⁢η+γ⁢ξ+ζ

η represents the internal dependent variable, ξ represents the external dependent variable, and ζ represents the residual term of the structural equation. The measurement model is shown in Eqs 3, 4:


(3) $$X=\wedge_{x}\,\text{ξ}+\delta$$

(4) $$Y=\wedge_{y}\,\eta +\varepsilon$$

By testing the validity of the questionnaire, the validity rate is about to 86%, indicating that the validity is good. Questionnaires can be used for practical survey applications. A total of 100 questionnaires are distributed to colleges and universities in a certain place. After the survey, 92 questionnaires are returned. Among them, there are about 79 valid questionnaires.

The questionnaire data are tested for reliability, and the value of Cronbach’s α is 0.821. The data indicated a high internal consistency among the various items of the questionnaire. In addition, through the validity test of the questionnaire, the validity is about to 86%, indicating that the validity is good. Questionnaires can be used in practical survey applications. The optimal construction effect of the structural equation model requires the sample size to be greater than 100. However, the main objects in the actual modeling process are college students. The limitation of model conditions can reduce the demand for samples and reduce the computational complexity of the model. Therefore, 100 questionnaires are distributed to a local college. After the survey, 92 questionnaires are collected. After the screening, 79 representative research subjects were selected, including college students of different majors, grades, genders, and age groups, and their questionnaire results were analyzed. A structural model of FAT mobilizing college students’ entrepreneurial mentality is established.

## Results

### Summary of Questionnaire Mean and Standard Deviation Data

**FIGURE 6:**
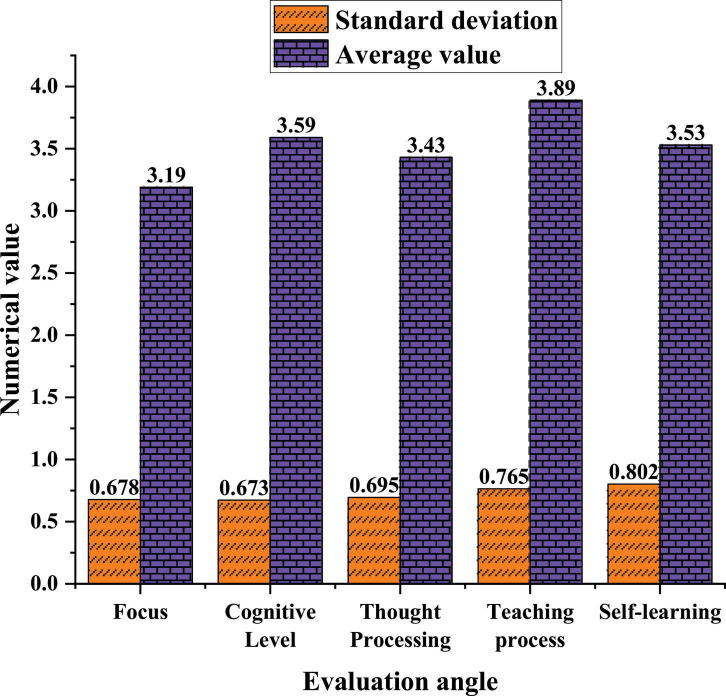
The standard deviation of data results.

Among the entrepreneurial intentions of FAT education, male students are much more than female students. Among the 79 valid questionnaires, about 56 male students, accounting for about 71%, and 23 female students, accounting for about 29%. SPSS software is used to count the results of the questionnaire data and to calculate the mean and standard deviation. Data are organized into statistical graphs. The five factors of concentration, cognitive level of deep learning, thought processing ability of deep learning, feeling of the teaching process, and autonomous learning process are abbreviated as concentration, cognitive level, thought processing, teaching process, and autonomous learning, as shown in Figure 6.

In Figure 6, at present, among the surveyed students in this university, the overall level of deep learning is not high, and none of them have reached the required status of each indicator. The average value of deep learning thought processing ability is 3.43, which does not reach the upper-middle level. The average student’s FAT study concentration is only 3.19, the lowest level. It shows that the students’ concentration level of FAT learning is insufficient. Therefore, teachers should guide and stimulate students’ learning satisfaction to promote the improvement of learning concentration. Secondly, students seriously lack initiative in the whole learning, they are passive learning, and there is no learning plan. Students’ self-evaluation ability is weak, and they cannot recognize their own problems in time and make corrections. For this state of learning, deeper learning needs to continue to be strengthened.

### Intrinsic Dimension Data Analysis of Deep Learning in Film and Television Education

**FIGURE 7:**
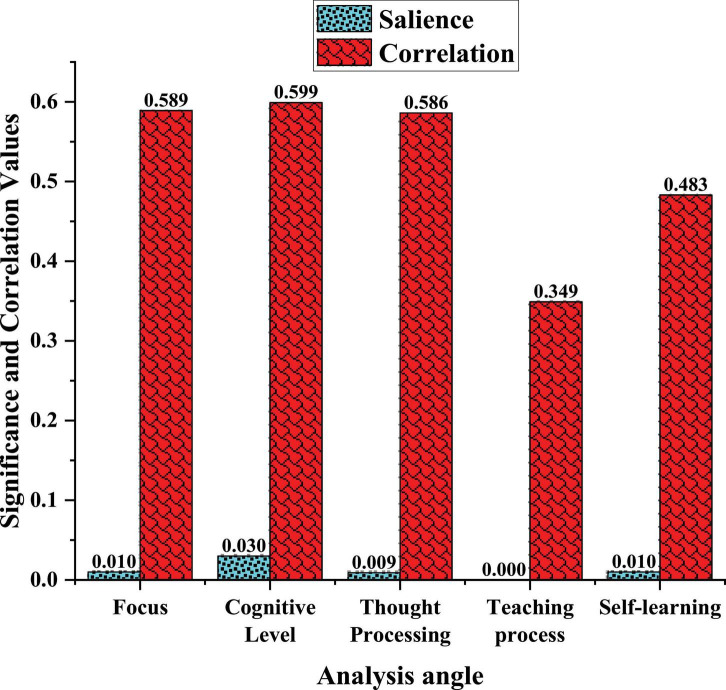
Multi-dimensional correlation analysis.

After the average and standard deviation of the data are analyzed, the next part analyzes and discusses the correlation between each data and the deep learning of FAT education. In addition, its significance is further investigated. The correlation between the five factors and the deep learning of FAT is judged to determine whether it is related to the students’ FAT entrepreneurial intention and what kind of relationship this relationship is. The resulting data are shown in Figure 7.

In Figure 7, the significant value of learning concentration is 0.010, less than 0.05, and the correlation value is 0.589, indicating that learning concentration and FAT entrepreneurial intention are related and positively correlated. The significance value of the deep learning cognitive level is 0.030, which is less than 0.05, and the correlation value is 0.599. It shows that the cognitive level is positively correlated with the FAT entrepreneurial intention. The significant number of deep learning thought processing ability is 0.009, less than 0.05, and the correlation value is 0.586. It shows that ideological processing ability is positively correlated with FAT entrepreneurial intention. The significance value of the teaching process feeling is 0 less than 0.05, and the correlation value is 0.349. It shows that the teaching process is positively correlated with the FAT entrepreneurial intention. Finally, the significance value of the self-learning process is 0.010, less than 0.05, and the correlation value is 0.483, which can also indicate that it has a positive correlation with the FAT entrepreneurial intention. These five factors can improve students’ entrepreneurial intentions in FAT after good deep learning, which further illustrates the important influence of the quality of deep learning education in FAT on the entrepreneurial ability of the industry.

## Conclusion

At present, the scale of online FAT resources is constantly expanding, and the quality is uneven. In this environment, this study explores how college students can screen for good, positive films, make their own choices, and improve factors regarding innovation and entrepreneurial intentions. Firstly, the phenomenon of shallow learning in current education and training is analyzed, and the concept of deep learning is expounded. Secondly, the impact of FAT education and job quality on the shaping of college students’ values is analyzed. Based on the FAT entrepreneurial intention of college students and the influencing factors of deep learning, a questionnaire is designed. After distributing and recycling the questionnaires, the survey results are processed, studied, analyzed, and discussed. The five factors of FAT learning concentration, deep learning cognitive level, deep learning thinking processing ability, teaching process feeling, and self-learning process all positively impact FAT entrepreneurial intention. The data shows the importance of deep learning in the FAT field to cultivate college students’ entrepreneurial intentions and reflects the same influence laws in other industries. These conclusions provide a certain direction for colleges and universities to cultivate talents in the FAT industry. However, some deficiencies still exist. The biggest limitation is that the number of data samples in the questionnaire is small, which reduces the reliability of the results of the questionnaire analysis. In the follow-up survey, the number of universities and research objects will be further expanded.

## Data Availability Statement

The raw data supporting the conclusions of this article will be made available by the authors, without undue reservation.

## Ethics Statement

The studies involving human participants were reviewed and approved by the Dalian Maritime University Ethics Committee. The patients/participants provided their written informed consent to participate in this study. Written informed consent was obtained from the individual(s) for the publication of any potentially identifiable images or data included in this article.

## Author Contributions

All authors listed have made a substantial, direct, and intellectual contribution to the work, and approved it for publication.

## Conflict of Interest

The authors declare that the research was conducted in the absence of any commercial or financial relationships that could be construed as a potential conflict of interest.

## Publisher’s Note

All claims expressed in this article are solely those of the authors and do not necessarily represent those of their affiliated organizations, or those of the publisher, the editors and the reviewers. Any product that may be evaluated in this article, or claim that may be made by its manufacturer, is not guaranteed or endorsed by the publisher.
